# P028 Probable giant cell arteritis presenting with cranial neuropathy and overlapping features of Tolosa–Hunt syndrome

**DOI:** 10.1093/rap/rkag113.058

**Published:** 2026-11-03

**Authors:** Maria Khan, Claire Masih

**Affiliations:** Ulster Hospital, South Eastern Health and Social Care Trust, Belfast, United Kingdom; Ulster Hospital, South Eastern H&SCT, Belfast, United Kingdom

## Abstract

**Introduction:**

Giant cell arteritis (GCA) is a large-vessel vasculitis affecting individuals over 50 years of age. It typically presents with headache, jaw claudication, and visual disturbance, but atypical presentations with cranial neuropathies may occur. Tolosa–Hunt syndrome is a rare idiopathic granulomatous inflammation of the cavernous sinus or orbital apex, presenting with ophthalmic nerve dysfunction and pain. Both conditions are corticosteroid responsive and may mimic each other clinically. We report a 71-year-old woman presenting with progressive cranial pain and evolving third cranial nerve palsy, highlighting diagnostic uncertainty between probable GCA and Tolosa–Hunt syndrome, with complete recovery following corticosteroid therapy.

**Case description:**

A 71-year-old woman presented with a 12-day history of progressive right-sided otalgia, described as sharp and stabbing, which evolved into severe right parietal and occipital scalp tenderness with marked allodynia and sleep disturbance. She had no jaw claudication, visual symptoms, polymyalgia rheumatica features, fever, or constitutional symptoms. Her medical history included hypertension, severe osteoporosis, osteoarthritis, and previous sigmoid colon carcinoma treated with colectomy.

Examination demonstrated right scalp tenderness with palpable, non-tender temporal arteries and a normal cranial nerve and ocular examination. Investigations showed an ESR of 45 mm/hr, CRP of 12 mg/L, and a Southend GCAPS score of 12, indicating intermediate risk for GCA. Given the atypical presentation, diagnostic uncertainty, and severe osteoporosis, corticosteroids were initially deferred while urgent CT brain and temporal artery ultrasound were arranged.

Over the next five days, she developed nausea, vomiting, and generalized headache with mild visual blurring. At that stage, her CT brain and ophthalmological assessment were unremarkable. After two days, she re-presented with binocular diplopia and persistent cranial pain. Her examination revealed a left pupil-sparing complete third cranial nerve palsy. She was treated with intravenous methylprednisolone followed by oral prednisolone 60 mg daily, resulting in rapid resolution of pain.

Temporal artery ultrasound performed one day after steroid initiation and temporal artery biopsy performed nine days later were both negative. MRI brain without contrast showed no evidence of cavernous sinus or orbital apex inflammation, while CT angiography identified incidental aneurysm unrelated to her symptoms. Neurology considered Tolosa–Hunt syndrome and cervico-occipital neuralgia, and occipital nerve blocks were administered.

Her symptoms improved dramatically with corticosteroid therapy. Ptosis resolved first, followed by gradual recovery of ocular movements and complete resolution of diplopia. By two months, neurological examination was normal with full visual recovery. She remains well on a tapering prednisolone regimen with ongoing follow-up.

**Discussion:**

This case highlights a complex diagnostic overlap between probable giant cell arteritis and Tolosa–Hunt syndrome presenting with cranial neuropathy. The initial presentation of isolated ear pain and scalp allodynia without headache or systemic symptoms was atypical for GCA, delaying immediate treatment. Progressive evolution into diplopia with a pupil-sparing third nerve palsy suggested cranial nerve involvement due to inflammatory or ischemic pathology.

The raised ESR and intermediate GCAPS score supported a vasculitic process, although temporal artery ultrasound (TAUS) and biopsy were non-diagnostic. However, TAUS was performed only one day after commencement of steroids so would have expected it to be positive if it was GCA. Clinically, neurology considered Tolosa-Hunt syndrome a better fit for our patient’s symptoms than GCA. However, the fact that her MRI was performed without contrast meant less radiologic certainty.

Both GCA and Tolosa–Hunt syndrome are highly corticosteroid responsive, making therapeutic response non-specific. In this case, rapid improvement following steroid therapy, along with complete neurological recovery, supports an inflammatory etiology. The absence of definitive imaging or histological confirmation leaves the final diagnosis unresolved between probable GCA and Tolosa–Hunt syndrome with overlapping features.

This case emphasizes the limitations of relying on a single diagnostic modality in evolving cranial neuropathies. Instead, serial clinical assessment, risk scoring systems, and response to therapy remain essential in guiding management. Importantly, early steroid therapy likely prevented further neurological complications despite diagnostic uncertainty.

**Key learning points:**

1. Giant cell arteritis may present atypically with isolated cranial pain, ear pain, or scalp allodynia without classical headache or systemic features. Clinicians should maintain a high index of suspicion even in the absence of typical symptoms, particularly in older adults with elevated inflammatory markers.

2. Cranial neuropathies, including pupil-sparing third cranial nerve palsy, can occur in GCA and may mimic other inflammatory or ischemic conditions. These presentations require urgent evaluation due to the risk of irreversible neurological and visual complications.

3. Tolosa–Hunt syndrome is an important differential diagnosis in patients with painful cranial neuropathies. Although classically associated with cavernous sinus inflammation on MRI, imaging may be normal early in the disease course, and diagnosis remains largely clinical after exclusion of alternative causes.

4. Early MRI of the brain with contrast is useful in evaluating Tolosa-Hunt syndrome. However, normal imaging does not rule out inflammatory cranial neuropathies.

5. Corticosteroid therapy is highly effective in both GCA and Tolosa–Hunt syndrome. However, treatment prior to diagnostic imaging or biopsy may reduce the sensitivity of these investigations and complicate definitive diagnosis. Despite this, treatment should not be delayed when clinical suspicion is high.

6. Temporal artery ultrasound and biopsy remain important diagnostic tools in suspected GCA, but their interpretation must be integrated with clinical context, especially when performed after steroid initiation. Negative results do not exclude disease in atypical or partially treated cases.

7. Serial neurological examination is critical, as evolving symptoms may clarify diagnosis over time. In this case, progression from focal ear pain to cranial neuropathy highlighted disease evolution.

8. Ultimately, overlapping inflammatory cranial syndromes may be clinically indistinguishable at presentation. In such cases, a pragmatic diagnosis of probable inflammatory cranial vasculopathy and prompt corticosteroid treatment may be justified to prevent irreversible neurological damage.

